# An *ETS2* Enhancer Variant May Modulate Gene Expression and Contribute to Defining a Genetic Risk Profile for SLE Susceptibility

**DOI:** 10.3390/genes16121462

**Published:** 2025-12-08

**Authors:** Andrea Latini, Giada De Benedittis, Chiara Morgante, Carlo Perricone, Fulvia Ceccarelli, Fabrizio Conti, Giuseppe Novelli, Cinzia Ciccacci, Paola Borgiani

**Affiliations:** 1 UniCamillus, Saint Camillus International University of Health Sciences, 00131 Rome, Italy; cinzia.ciccacci@unicamillus.org; 2 Department of Biomedicine and Prevention, Genetics Section, University of Rome Tor Vergata, 00133 Rome, Italy; dbngdi01@uniroma2.it (G.D.B.); chiaramorgante89@gmail.com (C.M.); novelli@med.uniroma2.it (G.N.); borgiani@med.uniroma2.it (P.B.); 3 Rheumatology, Department of Medicine and Surgery, University of Perugia, Piazzale Giorgio Menghini, 1, 06129 Perugia, Italy; carlo.perricone@gmail.com; 4 Lupus Clinic, Rheumatology, Department of Internal Medicine, Sapienza University of Rome, 00161 Rome, Italy; fulviaceccarelli@gmail.com (F.C.); fabrizio.conti@uniroma1.it (F.C.)

**Keywords:** systemic lupus erythematosus, ETS2, single-nucleotide variant, genetic risk score

## Abstract

**Background/Objectives**: Systemic lupus erythematosus (SLE) is a multifactorial autoimmune disease strongly influenced by genetic factors. Genome-wide association studies (GWASs) have identified numerous non-coding susceptibility loci, but their functional roles remain poorly understood. The single-nucleotide variant (SNV) rs2836882, located in an enhancer near the *ETS2* proto-oncogene, has been implicated in immune regulation, though its contribution to SLE is unclear. **Methods**: We analyzed rs2836882 in 246 Italian patients with SLE and 216 matched controls using TaqMan genotyping. A weighted genetic risk score (wGRS) combining rs2836882 with other known SLE variants was calculated. *ETS2* mRNA expression was quantified by RT-qPCR in PBMCs from 60 individuals, and in silico analyses assessed the variant’s functional context. **Results**: The rs2836882 risk allele was significantly associated with SLE (OR = 1.54, *p* = 0.02). Patients showed a markedly higher wGRS than controls (*p* < 0.00001), confirming an additive genetic burden. In silico data indicated that rs2836882 lies within an active enhancer region (H3K4me1/H3K27ac+) containing PU.1 binding motifs and functions as an expression quantitative trait locus (eQTL) for *ETS2*. Expression analysis demonstrated that carriers of the risk allele exhibited significantly increased *ETS2* expression compared to non-carriers (*p* = 0.002) in both groups. **Conclusions**: In conclusion, rs2836882 is a functional regulatory variant that enhances *ETS2* transcription and contributes to increased SLE susceptibility. These findings establish a mechanistic link between a non-coding GWAS locus and disease risk, emphasizing the role of regulatory variants in autoimmune pathogenesis and supporting the integration of functional non-coding variants into genetic risk models for improved patient stratification.

## 1. Introduction

Systemic lupus erythematosus (SLE) is a chronic and relapsing autoimmune disorder, characterized by the production of autoantibodies against intracellular components, nucleic acids, and cell surface antigens. The disease exhibits a wide clinical variety, affecting multiple organ systems including the skin, kidneys, lungs, heart, and central nervous system. The pathogenesis of SLE is complex and results by an interplay of genetic susceptibility and environmental factors, which jointly influence disease onset, progression, and clinical heterogeneity [1].

Numerous studies have demonstrated that genetic factors play an essential role in SLE development [2]. The identification of genetic variants associated with SLE can help to understand the pathogenic mechanisms underlying the disease. Although many of these genetic variants are located in genes encoding proteins involved in key pathogenic pathways, numerous susceptibility loci identified through genome-wide association studies (GWASs) are located in intergenic regions [3,4].

Functional genomics studies are highlighting the crucial role of genetic variants located in non-coding regions in modulating gene expression [5,6]. For example, a recent study has demonstrated that an intergenic locus on chromosome 21q22, described to be associated with multiple inflammatory diseases, including inflammatory bowel disease, ankylosing spondylitis, and Takayasu arteritis, is located in an enhancer element [7]. Fine mapping and functional analyses have identified the transcription factor *ETS2*, located approximately 200 kb from this locus, as a key effector gene regulated by it. In particular, the single-nucleotide variant (SNV) rs2836882 has been shown to improve chromatin accessibility and promote *ETS2* expression in monocytes and macrophages through allele-specific binding of the pioneer transcription factor PU.1 [7].

ETS2 is a member of the ETS family of transcription factors, known for its involvement in various cellular mechanisms such as proliferation, differentiation, and apoptosis. Increasing evidence indicates that ETS2 also participates in the regulation of immune response cells, particularly in monocytes and macrophages [8]. In these cells, ETS2 acts as a central regulator of inflammation by controlling the expression of a broad network of genes involved in cytokine production (including IL-6, IL-1β, and TNF-α), chemotaxis, and phagocytosis [9]. Overexpression of *ETS2* induces inflammatory pathways, closely emulating the transcriptional profile observed in macrophages of inflammatory diseases such as Crohn’s disease [10]. Consistently, a higher expression of *ETS2* has been observed in patients with asthma [11] and inflammatory bowel disease [12].

This study aims to investigate the association between the rs2836882 SNV, located within an enhancer region of the *ETS2* gene, and susceptibility to SLE in an Italian cohort. Additionally, it assesses the impact of rs2836882 on *ETS2* gene expression in PBMCs and incorporates this variant into a comprehensive genetic risk profile, combining it with other known SLE-associated polymorphisms.

## 2. Materials and Methods

### 2.1. Patient Recruitment

We retrospectively enrolled 246 Caucasian patients aged 18 years or older with SLE at the Lupus Clinic of the Rheumatology Unit, Sapienza University of Rome. All patients fulfilled the criteria of European League Against Rheumatism and American College of Rheumatology (ACR) for the classification of SLE [13]. Written informed consent was obtained from all participants, and the study protocol was approved by the Ethics Committee of the Policlinico Umberto I (protocol number 26/3/12/2015).

The study protocol included a comprehensive physical examination and blood sampling. Clinical and laboratory data were recorded using a standardized, electronically compiled form, which included demographic information, past medical history, comorbidities, and details of previous and concomitant treatments. Clinical and laboratory features were assessed using a dichotomous scoring system (present = 1; absent = 0). Patient characteristics are described in Table 1.

As a control group, 216 healthy, sex-, age- and ethnicity-matched individuals (43.9 ± 10.21 years; 83.3% females) were enrolled at the University of Rome Tor Vergata.

### 2.2. DNA Extraction and Genotyping

Genomic DNA was extracted from peripheral blood using standard procedures and the Qiagen Blood DNA Mini Kit. Patients and controls were analyzed for rs2836882 SNV using TaqMan assays on a 7500 Real-Time PCR System (Applied Biosystems). Known genotype samples (validated through direct sequencing) were included in each run as controls. For each subject, a weighted genetic risk score was calculated as the sum between the number of risk alleles and their corresponding odds ratio (OR), derived from previous association studies on this cohort [14,15,16,17]. The selected polymorphisms included rs2230926, rs6920220 (*TNFAIP3*), rs33980500 (*TRAF3IP2*), rs7574865 (*STAT4*), rs573775 (*ATG5*), and rs2836882 (analyzed in the present study). The cumulative weighted risk score for each participant was subsequently rounded to the nearest whole number. Subjects were grouped according to their final score, and the frequency of individuals within each risk-score category was determined for both patients with SLE and controls.

### 2.3. In Silico Analysis

The potential functional impact of the rs2836882 variant was assessed using HaploReg v4.2. Chromatin features, including enhancer-associated histone marks and DNase I hypersensitivity sites, were examined in immune-relevant cell types. GTEx eQTL data were queried to assess the influence of rs2836882 on *ETS2* expression in immune tissues. ETS2 cell-type-specific expression was explored using publicly available RNA-seq data from the DICE database (dice-database.org)

### 2.4. RNA Extraction and Expression Study

Thirty patients with SLE and thirty healthy controls were randomly selected from the first cohort for the expression study. Total RNA was isolated from peripheral blood mononuclear cells (PBMCs) using TRIzol reagent (Ambion, Foster City, CA, USA), followed by reverse transcription using the High-Capacity cDNA Reverse Transcription Kit (Applied Biosystems, Waltham, MA, USA). Quantitative real-time PCR (qRT-PCR) was performed using the SYBR Green method (Applied Biosystems) with the following primers for ETS2: Fw:5′-CTGACTTTGTGGGTGACAT-3′; RV:5′-CTGTTAATCCAATGAGGAACG-3′. β-ACTIN was used as the endogenous control gene, with the following primers: forward: 5′-AAGATGACCCAGATCATGTTTGAGACC-3′; reverse: 5′-ATCCTGCGTCTGGACCTGGCGT-3′. Reactions were run on the 7500 Real-Time PCR System. Gene expression levels were quantified using the 2^−ΔΔCt^ method and reported as relative expression values.

### 2.5. Statistical Analysis

The Hardy–Weinberg equilibrium (HWE) of this polymorphism was assessed in both groups using Pearson’s χ^2^ test. Differences in genotypic frequencies between cases and controls were analyzed using Pearson’s χ^2^ test. Heterozygous and variant homozygous individuals were grouped together in a dominant model. Odds ratios (ORs) with 95% confidence intervals (CIs) were calculated. A *p*-value ≤ 0.05 was considered statistically significant. Gene expression data were analyzed in triplicate, and results were expressed as the mean ± standard error (SE). Comparisons between groups were performed using the ANOVA test. All statistical analyses were conducted using SPSS version 27 (IBM Corp., Armonk, NY, USA). All graphs were generated using GraphPad Prism 10 (GraphPad Software, Boston, MA, USA).

## 3. Results

### 3.1. Genotyping Study

Firstly, we investigated the genotype distribution of the rs2836882 SNV in a cohort of 246 patients with SLE and 216 healthy controls. No deviations from the Hardy–Weinberg equilibrium were observed for this polymorphism. The distribution of genotype frequencies and the comparisons between cases and controls are reported in Table 2. As is shown, rs2836882 SNV was associated with increased susceptibility to SLE. In particular, the variant allele was significantly more present in patients than in controls, with an OR = 1.54 [95% CI: 1.07–2.23] and *p* = 0.02.

The genotype distribution observed in our control group was consistent with frequencies reported in the gnomAD database for the non-Finnish European population (GG: 53.3%, GA: 39.4%, AA: 7.3%). Furthermore, when comparing the genotype distribution in our patients with SLE to that reported in gnomAD, the difference was statistically significant (*p* = 0.005), further supporting the association of this variant with SLE susceptibility.

**Figure 1:**
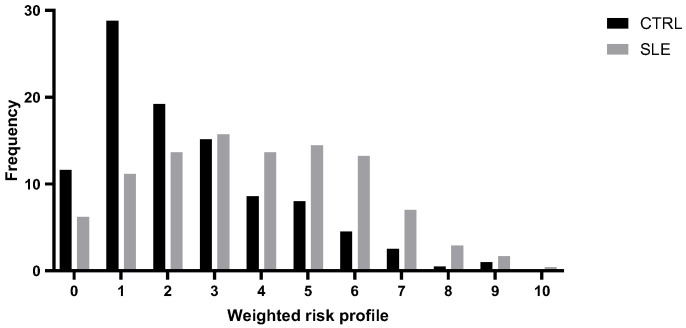
Distribution of the weighted risk profile in patients with systemic lupus erythematosus (SLE) and healthy controls (CTRLs).

To examine the cumulative genetic risk associated with SLE, we constructed a genetic risk profile by integrating the rs2836882 polymorphism with other risk genetic variants previously identified in this same cohort [14,15,16,17]. Specifically, we considered as genetic risk factors, besides the rs2836882, two polymorphisms in the *TNFAIP3* gene (rs2230926 and rs6920220), one polymorphism in the *TRAF3IP2* gene (rs33980500), one polymorphism in the *STAT4* gene (rs7574865), and one polymorphism in the *ATG5* gene (rs573775). For each subject, the individual weighted genetic risk profile was calculated as the sum of the products of the number of risk alleles and their respective ORs for SLE susceptibility, derived from our cohort data (Figure 1).

This analysis highlighted a normal distribution of risk profile in both groups, with statistically significant differences. As expected, individuals in the low-risk class (≤2) were significantly more prevalent in the control group (*p* = 0.00001), while high-risk classes (≥5) were overrepresented among patients with SLE (*p* = 0.00001).

### 3.2. In Silico Analysis

To further investigate the potential functional implications of the rs2836882 variant, we performed an in silico analysis using the HaploReg v4.2 tool. This analysis revealed that rs2836882 is located within an intronic region of the *ETS2* gene, enriched with enhancer-associated histone marks (H3K4me1, H3K27ac) in immune-related cell types. Furthermore, this SNV is located in a DNase I hypersensitive site, indicative of open chromatin, and is predicted to alter a specific binding site for the transcription factor PU.1, already known for its role in specific regulation in monocytes and macrophages. GTEx data further indicate that rs2836882 acts as an eQTL for *ETS2* in immune tissues, further supporting its role as a transcriptional modulator. Moreover, this variant is also in high linkage disequilibrium (r^2^ ≥ 0.85) with at least three other SNPs mapped to putative regulatory elements. To explore the potential cell-type-specific regulation of *ETS2*, we examined publicly available expression datasets. Data from the DICE database showed that *ETS2* expression is predominantly enriched in monocytes and monocyte-derived macrophages, with markedly lower levels in lymphoid lineages. Moreover, inspection of single-cell RNA-seq PBMC data showed that *ETS2* expression is concentrated in monocyte clusters.

### 3.3. Expression Study

**Figure 2:**
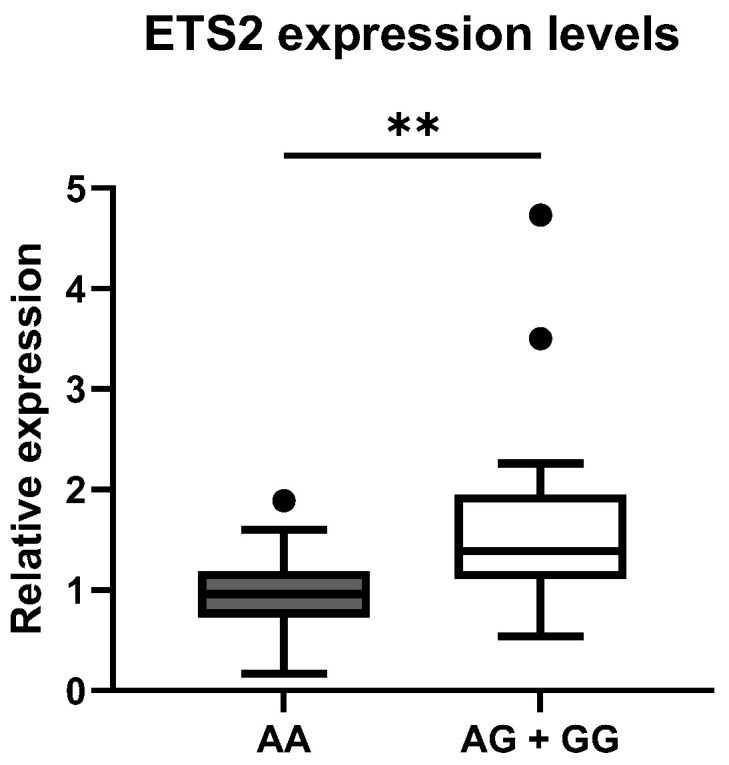
Distribution of mean expression levels of ETS2 between the two genotypic classes for the polymorphism rs2836882. ** *p*-value < 0.01.

To confirm the possible association between rs2836882 and the different expression levels of the *ETS2* gene, we evaluated the expression levels of this gene by RT-qPCR in PBMCs from 30 patients with SLE and 30 healthy CTRLs, randomly selected from the larger genomic cohort. We compared the distribution of the mean values in the different genotypic classes. As is shown in Figure 2, the rs2836882 polymorphism variant allele in the *ETS2* enhancer region was associated with a higher expression of this gene. In particular, carriers of the variant allele (AG and AA genotypes) showed a significantly higher *ETS2* expression compared with patients with the homozygous wild-type genotype (GG) (*p* = 0.002).

**Figure 3:**
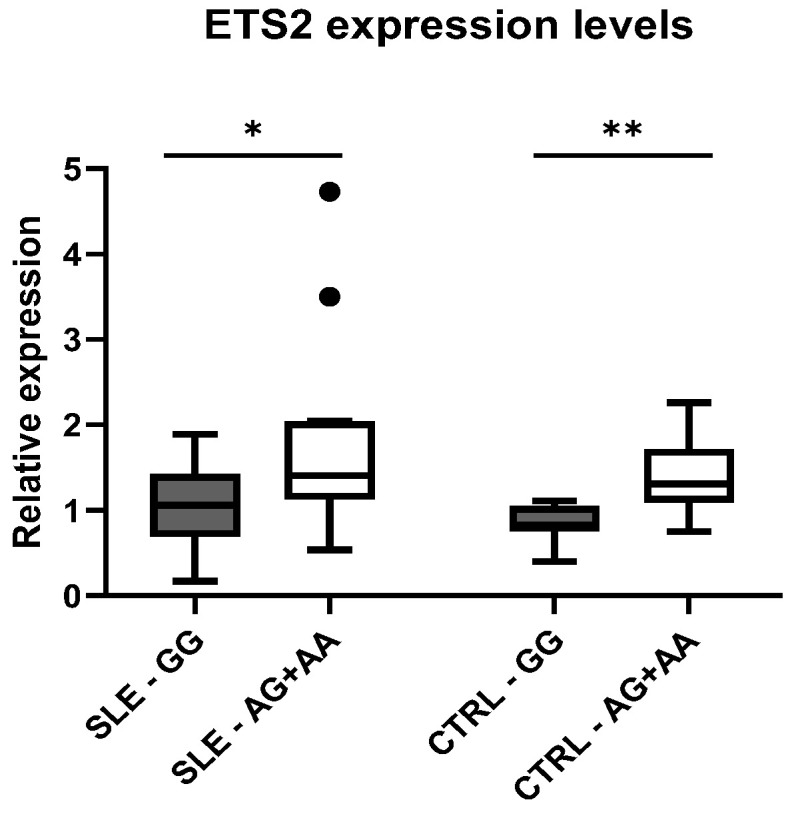
Distribution of mean expression levels of ETS2 between the genotypic classes for the polymorphism rs2836882 in patients with SLE and CTRLs. * *p*-value < 0.05, ** *p*-value < 0.01.

The difference in expression between genotypic classes was also verified independently in cases and controls. In fact, we observed higher levels of *ETS2* expression in subjects carrying the variant, both in patients with SLE (*p* = 0.01) and in healthy controls (*p* = 0.003), compared to wild-type individuals (Figure 3).

**Figure 4:**
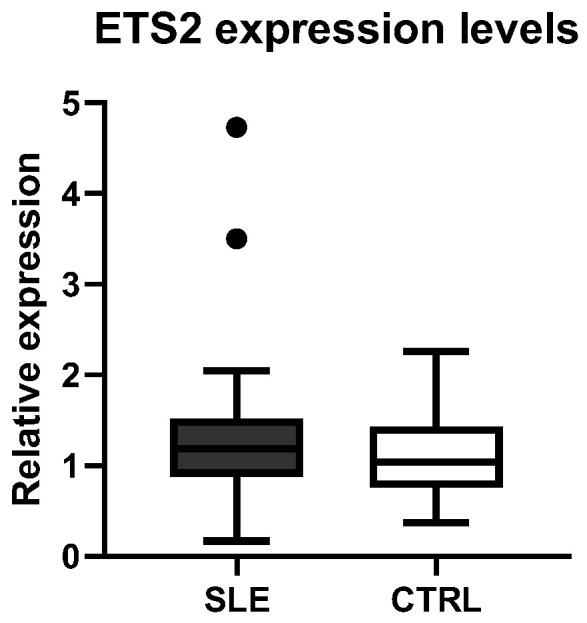
Comparison of ETS2 expression levels between healthy controls (CTRLs) and patients with SLE.

Moreover, we compared the *ETS2* expression levels between patients with SLE and healthy CTRLs, but no significant differences were observed between the two phenotypic groups (Figure 4).

In addition, we performed exploratory analyses to assess whether *ETS2* expression was associated with specific clinical manifestations of SLE. While no correlation was found with the SLEDAI (Systemic Lupus Disease Activity Index) score, patients with pericarditis showed a higher *ETS2* expression compared with those without this manifestation (*p* = 0.03).

## 4. Discussion

This study explored the potential role of the rs2836882 SNV, located in an enhancer region of the *ETS2* gene, and the susceptibility to SLE. Our results showed a statistically significant association between the presence of the rs2836882 variant allele and an increased risk of SLE in a well-characterized Italian cohort. This association was further supported by comparison with allele frequencies in the European population reported in the gnomAD database. These results are consistent with previous GWASs, which have identified rs2836882 as a regulatory variant involved in other autoimmune diseases [7]. In particular, rs2836882 is located in a region enriched for histone modifications typical of active enhancers, and it overlaps with DNase I hypersensitive sites across multiple immune cell types. High-resolution chromatin conformation studies have revealed a monocyte-specific physical interaction between this enhancer region and the *ETS2* promoter, indicating a potential cis-regulatory mechanism [18].

Functional annotation and in silico predictions suggest that rs2836882 influences binding motifs for key transcription factors, particularly PU.1, a pioneer factor essential for myeloid cell differentiation and function. The SNV is located within a highly conserved 17 bp AP-1-like motif, homologous to the X2 box of HLA-DRA promoters, and known to interact with factors of the ATF/CREB family. Moreover, comparative analyses between species reveal that the sequence surrounding rs2836882 is highly conserved in mammals, suggesting the potential for future functional studies in vivo using animal models [19].

Further transcriptomic analyses from the FANTOM5 consortium have revealed enhancer activity across the entire intergenic region between ETS2 and PSMG1 genes, including transcription of long non-coding RNAs (ETS2-AS1, LINC02940, LINC02943), reinforcing the idea that this locus may function as a super-enhancer with complex regulatory potential [20]. Notably, a recent GWAS from the UK Biobank also reported an association between rs2836882 and circulating neutrophil counts, further highlighting the link of this variant to immune system regulation [21].

To explore the functional impact of rs2836882 in our cohort, we performed gene expression analyses. We observed that carriers of the variant allele showed a significantly higher level of *ETS2* mRNA in PBMCs, both in patients with SLE and healthy controls. This correlation suggests that rs2836882 acts as a cis-eQTL, independently of the disease. Our results agree with GTEx data, which report similar eQTL effects in monocytes. Interestingly, no overall difference in *ETS2* expression was observed between patients with SLE and controls. This indicates that disease status does not globally alter *ETS2* transcription in circulating PBMCs. However, it should be noted that most patients with SLE were receiving glucocorticoids or immunosuppressive therapy, which may modulate inflammatory gene expression. Therefore, treatment-related effects might have attenuated potential disease-specific differences in *ETS2* levels. Moreover, this lack of difference likely reflects the cellular heterogeneity of PBMCs, where *ETS2* expression is mainly restricted to monocytes and macrophages. In SLE, *ETS2* may be modulated in a subset-specific or an activation-dependent manner, rather than being uniformly upregulated. Indeed, *ETS2* expression is known to respond dynamically to type I interferons and Toll-like receptor signaling, pathways that are aberrantly activated in SLE [22]. Therefore, the pathogenic effect of *ETS2* may emerge only in specific inflammatory or tissue contexts. The rs2836882 variant has previously been implicated in autoimmune and inflammatory diseases such as Crohn’s disease and ulcerative colitis [10,12], where it enhances *ETS2* transcription in monocytes. Our findings extend this association to SLE, suggesting a shared regulatory axis across distinct immune-mediated disorders.

*ETS2* has been increasingly recognized as a central transcriptional regulator of inflammation in myeloid cells. It directly binds to promoters of pro-inflammatory cytokines, such as IL-6, IL-1β, and TNF-α, thereby amplifying innate immune signaling through MAPK and NF-κB pathways [10,22]. *ETS2* overexpression has been shown to increase the number of reactive oxygen species and promote macrophage phagocytic capacity, while also regulating genes involved in cell migration and tissue infiltration [9,10]. Conversely, *ETS2* silencing suppresses inflammatory cytokine expression and attenuates macrophage activation [22]. Given the centrality of these processes in SLE pathogenesis, the enhanced *ETS2* expression associated with rs2836882 may contribute to the immune imbalance and tissue damage observed in SLE, particularly in monocyte/macrophage subsets

By integrating the rs2836882 polymorphism with other genetic variants previously identified in this same cohort, our study developed a genetic profiling model that demonstrates a significant increase in the risk of developing SLE with the number of risk alleles. As is shown in Figure 1, patients with SLE exhibit a higher genetic risk score compared to those in the control group, suggesting that the presence of specific alleles at six genetic loci confers a greater susceptibility to the disease, resulting in a shift to the right of the distribution of scores. The results of the Pearson χ^2^ test indicate a significant difference in the frequency of individuals classified as being at low risk (≤2 risk alleles) and high risk (≥5 risk alleles) between cases and controls. The progressive increase in disease susceptibility with higher wGRS categories supports the additive nature of these variants and their potential utility for patient stratification. Genetic risk profiling thus represents a valuable approach to evaluate the cumulative effect of multiple genotypes and their association with complex diseases. Although this model is exploratory and derived from a single population, it illustrates how incorporating functional non-coding variants into polygenic frameworks can enhance predictive precision. Future improvements may include the inclusion of additional risk loci identified by large-scale GWASs, integration of transcriptomic or epigenetic markers, and validation in independent and multiethnic cohorts. Lastly, multi-locus genetic models could support individualized risk estimation and early diagnosis, and potentially inform personalized therapeutic strategies in autoimmune diseases such as SLE.

This study has several limitations. First, *ETS2* expression was assessed in bulk PBMCs, which may mask cell-type-specific effects, particularly within monocytes or macrophages, where *ETS2* should be mainly expressed. Second, although the observed association between rs2836882 and *ETS2* expression support a functional relationship, as well as in silico analyses, direct experimental validation of the enhancer activity was not performed. Lastly, although the cohort was homogeneous and well characterized, replication in independent and multiethnic populations would strengthen our findings and help determine whether the impact of rs2836882 on *ETS2* regulation and SLE susceptibility is consistent across different genetic backgrounds.

## 5. Conclusions

In conclusion, our study describes, for the first time, the association between the rs2836882 variant, located in an *ETS2* enhancer, and susceptibility to SLE. This variant appears to correlate with an increased *ETS2* expression in PBMCs, supporting its functional role as a cis-eQTL that is likely mediated by altered transcription factor binding and chromatin accessibility, and highlighting the importance of non-coding regulatory variants in the pathogenesis of autoimmune diseases. Although further functional studies and validation in larger and independent cohorts are needed, our findings provide new insights into the genetic and transcriptional mechanisms underlying SLE.

## Figures and Tables

**Table 1:** Clinical and laboratory data of the patients with SLE.

	Patients with SLE Genomic Study (n = 246)	Patients with SLE Expression Study (n = 30)
Age (years ± SD)	43.84 ± 11.43	47.37 ± 12.94
Age at onset (years ± SD)	33.53 ± 12.33	33.20 ± 14.11
Disease duration (years ± SD)	13.21 ± 8.49	14.50 ± 8.32
Sex (% female)	89.0%	93.3%
Musculo-skeletal involvement *	67.1%	40.0%
Photosensitivity	63.1%	71.4%
Malar rash *	57.5%	33.3%
Aphthous ulcers	18.2%	14.3%
Pericarditis	20.5%	20.0%
Pleurisy	16.4%	6.7%
Nephritis	29.5%	23.3%
Anemia	41.7%	46.7%
Leucopoenia	42.2%	30.8%
Thrombocytopenia	17.1%	16.7%
ANA	96.2%	100%
Anti-dsDNA *	69.7%	50.0%
Anti-Sm	14.6%	21.4%
Anti-RNP	17.4%	25.0%
Anti-Ro/SSA	33.8%	46.4%
Anti-La/SSB	15.9%	17.9%
Anti-CL IgG or IgM	38.0%	44.8%
Anti-β2GPI IgG or IgM	22.9%	20.0%
LAC	28.1%	33.3%
C3 (below normal values)	50.3%	31.0%
C4 (below normal values)	46.2%	37.9%

Quantitative data are expressed as mean and standard deviation (SD); dichotomous data are ex-pressed as a percentage. SLE: systemic lupus erythematosus; ANA: antinuclear antibodies; anti-dsDNA: anti-double-stranded DNA; anti-RNP: ribonucleoprotein antibodies; anti-CL: anticardiolipin antibodies; anti-β2GPI: anti-β2 glycoprotein I; LAC: lupus anticoagulant; C3: complement 3; C4: complement 4. * Statistically significant difference between groups (*p* < 0.05).

**Table 2:** Case-control association analysis between rs2836882 SNV and systemic lupus erythematosus (SLE).

rs2836882				(GG vs. AG + AA)	
	GG	GA	AA	P	OR (95% CI)
**SLE**	109 (44.3%)	111 (45.1%)	26 (10.6%)	**0.02**	1.54 (1.07–2.23)
**Controls**	119 (55.1%)	82 (38.0%)	15 (6.9%)		

P = *p*-value evaluated by Pearson’s *χ*^2^ test; OR (95% CI) = odd ratio with 95% confidence interval.

## Data Availability

No new data were created or analyzed in this study. Data sharing is not applicable to this article.

## Abbreviations

The following abbreviations are used in this manuscript:

SLE	Systemic lupus erythematosus
SNV	Single-nucleotide variant
wGRS	Weighted genetic risk score
GWASs	Genome-wide association studies
eQTL	Expression quantitative trait locus
ACR	American College of Rheumatology
PBMCs	Peripheral blood mononuclear cells
OR	Odds ratio
SE	Standard error
CI	Confidence interval
CTRLs	Healthy controls
